# Response to: Comment on “New Alternatives for Autoimmune Disease Treatments: Physicochemical and Clinical Comparability of Biosimilar Etanercept”

**DOI:** 10.1155/2018/6156024

**Published:** 2018-02-21

**Authors:** Mariana P. Miranda-Hernández, Carlos A. López-Morales, Francisco C. Perdomo-Abúndez, Rodolfo D. Salazar-Flores, Nancy D. Ramírez-Ibanez, Nestor O. Pérez, Aaron Molina-Pérez, Jorge Revilla-Beltri, Emilio Medina-Rivero, Luis F. Flores-Ortiz

**Affiliations:** Unidad de Investigación y Desarrollo, Probiomed S.A. de C.V., Cruce de Carreteras Acatzingo-Zumpahuacán S/N, Colonia Los Shiperes, 52400 Tenancingo de Degollado, MEX, Mexico

This communication responds to the Letter to the Editor [1], where noticeably Messrs. Brian Hassett, Steven Vicik, and Brian Fitzpatrick from Pfizer Biotech contended that a particular glycan profile, presented in comparison to different profiles, does not correspond to the historical profile of the reference medicinal product of etanercept (Enbrel®), as it is exposed.

As published, Figure 3(b) from our scientific article “New Alternatives for Autoimmune Disease Treatments: Physicochemical and Clinical Comparability of Biosimilar Etanercept” contains different glycan profiles of Enbrel [2]. In particular, an Enbrel chromatogram revealed a higher relative abundance of late eluting species. Therefore, it is suggested by Hassett et al. [1] that this profile belongs instead to the etanercept biosimilar: Infinitam®, manufactured by Probiomed S.A. de C.V. Additionally, it is reasoned that the chromatogram pertaining to Infinitam is indistinguishable from the reference medicinal product, as its identity is presumed as an Enbrel profile [1].

Although it is noticed and scientifically supported that the strong comparability between Infinitam and Enbrel allows such a declaration of indistinguishable characteristics between products, to suggest a mismanagement of results or false statement in our published scientific article is inappropriate and totally unwarranted.

All authors have a strong scientific and proven track record with several refereed publications, patents, and technical conferences. All the authors have accepted responsibility for their contributions, ensuring originality and integrity of the results with data presented according to the journal requirements. Besides, the data was generated upon the use of standardized practices and procedures in Probiomed S.A. de C.V., a pharmaceutical company certified in Good Manufacturing Practices by Mexico's Federal Commission for Protection against Sanitary Risks (COFEPRIS), which is recognized as a National Regulatory Authority by the World Health Organization. We therefore found surprising that the scientific integrity of the paper, the reputation of the authors, and the Journal of Immunology Research are questioned.

Actually, the mistrusted glycan profile (Enbrel chromatogram in Figure 3 panel b of Miranda-Hernández et al. [2]) largely corresponds to the Enbrel reference standard profile included in the Letter to the Editor [1]. We present a reproduction of both profiles as Figure 1 of this letter.

The chromatograms have been labeled (a) and (b) for this letter.

As it can be seen, Figure 1 of this letter reveals correspondence between Enbrel profiles, as obtained by Miranda-Hernández et al. [2] and Hassett et al. [1]. Moreover, a similar profile was published by a third party assessment on Enbrel glycan heterogeneity [3] (Figure S-1 of Houel et al.'s “N- and O-Glycosylation Analysis of Etanercept Using Liquid Chromatography and Quadrupole Time-of-Flight Mass Spectrometry Equipped with Electron Transfer Dissociation Functionality”). Equivalence can be unquestionably stated among glycan profiles obtained by different laboratories using UPLC.

It is worth to notice that variations of the relative abundances within the major glycan structures along the historical manufacturing experience of Enbrel have been previously reported by different contributions, namely, Piña-Lara et al.'s “Characterization and Comparability of Etanercept Biosimilar” [4] and Schiestl et al.'s “Acceptable Changes in Quality Attributes of Glycosylated Biopharmaceuticals” [5]. The latter reported glycan variations based on the analysis of different batches, covering 5 years of commercial manufacture of Enbrel.

Further, on the Letter to the Editor 5047067 [1], it is contended that the glycan profile of the biosimilar product Infinitam revealed the presence of two additional glycan species that are not resolved in Enbrel. Specifically, triantennary glycans (A3G3F and A3G3FS1) that are reported as additional structures in the biosimilar product Infinitam. However, it is known that larger triantennary and tetraantenary glycans occur at the TNF-*α* receptor region of etanercept, particularly in Enbrel [3] (Figure 4b and Table S-1 of Houel et al.'s “N- and O-Glycosylation Analysis of Etanercept Using Liquid Chromatography and Quadrupole Time-of-Flight Mass Spectrometry Equipped with Electron Transfer Dissociation Functionality”). We therefore find that triantennary and tetraantenary glycan structures were misinterpreted as additional species in Infinitam by Hassett et al. [1].

This letter asserts that the accuracy of all the Enbrel chromatograms presented in our scientific contribution “New Alternatives for Autoimmune Disease Treatments: Physicochemical and Clinical Comparability of Biosimilar Etanercept” [2] is unquestionable. The results and conclusions are sustained on a state-of-the-art technology and supported by different studies published worldwide.

In summary, “New Alternatives for Autoimmune Disease Treatments: Physicochemical and Clinical Comparability of Biosimilar Etanercept” [2] presents an overview of the evidence of Infinitam biosimilarity, including physicochemical, functional, and clinical data. Besides, the high heterogeneity intrinsic to etanercept was described, as observed among batches of the reference medicinal product, Enbrel. Our findings revealed comparable profiles among products, thus evidencing that Infinitam is highly similar to Enbrel.

## Figures and Tables

**Figure 1:**
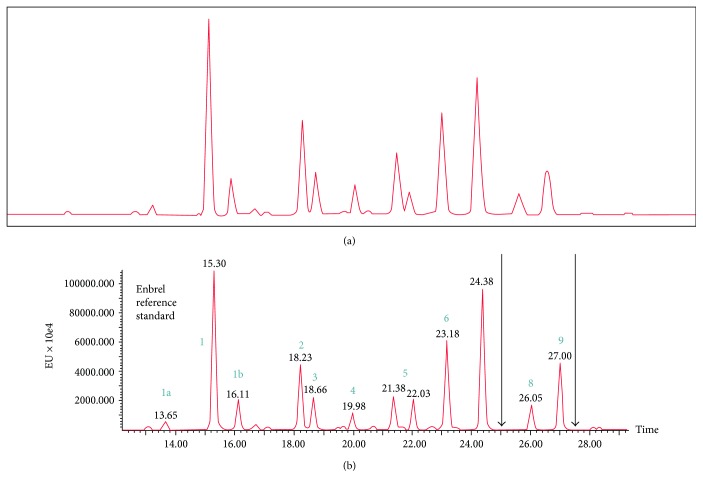
(a) Questioned Enbrel chromatogram included in Figure 3 panel b of Miranda-Hernández et al. [2] in comparison to (b) Pfizer's analysis by UPLC of Enbrel reference standard included in the Letter to the Editor 5047067 [1].
